# Predictors of Gait Speeds and the Relationship of Gait Speeds to Falls in Men and Women with Parkinson Disease

**DOI:** 10.1155/2013/141720

**Published:** 2013-06-05

**Authors:** Samuel T. Nemanich, Ryan P. Duncan, Leland E. Dibble, James T. Cavanaugh, Terry D. Ellis, Matthew P. Ford, Kenneth B. Foreman, Gammon M. Earhart

**Affiliations:** ^1^Program in Physical Therapy, Washington University in St. Louis, St. Louis, MO 63108, USA; ^2^Department of Physical Therapy, University of Utah, Salt Lake City, UT 84108, USA; ^3^Department of Physical Therapy, University of New England, Portland, ME 04103, USA; ^4^Department of Physical Therapy and Athletic Training, Boston University, Boston, MA 02215, USA; ^5^Department of Physical Therapy, School of Health Professions, University of Alabama at Birmingham, Birmingham, AL 35294, USA; ^6^Movement Science PhD Program, Physical Therapy, Anatomy & Neurobiology, and Neurology, Washington University School of Medicine, Program in Physical Therapy, Campus Box 8502, 4444 Forest Park Boulevard, St. Louis, MO 63108, USA

## Abstract

Gait difficulties and falls are commonly reported in people with Parkinson disease (PD). Reduction in gait speed is a major characteristic of Parkinsonian gait, yet little is known about its underlying determinants, its ability to reflect an internal reservation about walking, or its relationship to falls. To study these issues, we selected age, disease severity, and nonmotor factors (i.e., depression, quality of life, balance confidence, and exercise beliefs and attitudes) to predict self-selected (SELF), fast-as-possible (FAST), and the difference (DIFF) between these walking speeds in 78 individuals with PD. We also examined gender differences in gait speeds and evaluated how gait speeds were related to a retrospective fall report. Age, disease severity, and balance confidence were strong predictors of SELF, FAST, and, to a lesser extent, DIFF. All three parameters were strongly associated with falling. DIFF was significantly greater in men compared to women and was significantly associated with male but not female fallers. The results supported the clinical utility of using a suite of gait speed parameters to provide insight into the gait difficulties and differentiating between fallers in people with PD.

## 1. Introduction

Gait difficulties are one of the first problems reported in people with PD, indicating the onset of disability [1]. Parkinsonian gait is often slow and characterized by short shuffling steps, which may contribute to postural instability. As such, problems with walking are often accompanied by falling, which occurs in 40–70% of people with PD [2]. Fallers have higher incidences of skeletal fracture, social isolation, and reduction in exercise [3]. These consequences of a fall can in turn contribute to declines in gait and balance and lead to an additional increased risk of falling. Understanding these difficulties and developing criteria to identify people with PD who are at risk for falling are crucial to interrupt this devastating cycle of falls and injuries. 

Complex gait analyses use 3-dimensional kinematics to quantify the biomechanical and rhythmic impairments of gait in people with PD [4, 5]. These evaluations require sophisticated equipment and require higher level analysis. Within clinical settings, therapists currently use objective balance rating scales to measure gait and balance because they assess a wide range of postural and balance characteristics, are highly reproducible and predictive of falls [6]. However, clinical balance scales require trained raters and can be time consuming. As an alternative to the above methods, gait speed is a simple measurement to assess gait function and perhaps fall risk, in people with PD. Furthermore, gait speed can be easily obtained with only a measuring tape and stopwatch.

Self-selected gait speed (SELF) of people with PD has been associated with disability level (UPDRS) [7] as well as nonmotor characteristics, such as age and executive attention [8]. However, limited data is available to show which (and to what extent) nonmotor factors are associated with gait speeds. SELF was found to be both weakly [9] and moderately [10] correlated with Part II of the UPDRS, which assesses activities of daily living (ADL). Elbers et al. also showed that anxiety, depression, and reduced motivation were associated with community walking in 153 individuals with PD cohort [11]. These results suggest that gait may be in part determined by nonmotor behavior, such as lack of confidence about walking, depression, quality of life, or general aversive attitudes about activity. 

Fast-as-possible gait speed (FAST) is important to consider because, when compared to SELF, it measures one's ability to adapt gait speed to environmental demands [12]. However, it has not been studied frequently, mainly because it correlates with age and disease severity to a similar extent as comfortable gait speed [10, 13]. We believe that a third gait speed, DIFF (i.e., the difference between FAST and SELF), may uniquely provide insight into an individual's willingness and ability to change gait speed. Presumably, DIFF could be influenced by nonmotor factors, such as depression, quality of life, balance confidence, and/or exercise beliefs and attitudes. Thus, our first aim was to identify the predictors of three gait speeds SELF, FAST, and DIFF. We postulated that SELF and FAST would be best predicted by age and MDS-UPDRS, while DIFF would be better predicted by nonmotor factors.

SELF has further been studied as an important indicator of community ambulation [11] and fall risk [14] in people with PD. In the latter study, Paul et al. developed a powerful three-variable model which included self-selected gait speed to predict fallers with PD [14]. However, to our knowledge, no one has similarly shown how FAST and DIFF gait speed parameters are related to falling. The second aim of this work, therefore, was to assess the relationship between SELF, FAST, and DIFF gait speeds and fall history in individuals with PD. We hypothesized that, in comparison to SELF and FAST, DIFF would be a better discriminator between fallers and nonfallers because of its potential to reflect the ability to adapt walking speed to changes in environmental demands that could precipitate a fall.

## 2. Materials and Methods

### 2.1. Participants

Of 81 participants, full data sets from 78 individuals with “idiopathic” PD [15], recruited from the Washington University Movement Disorders Clinic, were analyzed. All participants were greater than 40 years of age, had a confirmed diagnosis of idiopathic PD from a neurologist, and were at a Hoehn and Yahr (H&Y) stage [16] between 1 and 4. Participants were included/excluded based on criteria defined previously [17]. All evaluations were performed during a single two-hour period during the “on” state, which was 1-2 hours after the administration of levodopa medication. Participants provided written consent after being screened for eligibility. This study was approved by the Human Research Protection Office at Washington University.

### 2.2. Clinical Evaluations and Questionnaires

All data were collected by a single assessor as part of a larger longitudinal study to monitor the outcomes of a cohort of people with PD [17]. Demographic information and medical history were obtained from each participant at the beginning of evaluation, including age, disease duration, medications, fall history, and exercise history. The full, revised Unified Parkinson's Disease Rating Scale (MDS-UPDRS) was administered to assess overall disease severity. Participants completed a battery of surveys and questionnaires during their visit. The Geriatric Depression Scale (GDS) was used to evaluate participants' emotional state and the Parkinson Disease Questionnaire-39 (PDQ-39) was used to indicate overall quality of life; high scores for the GDS are associated with greater levels of depression while high scores for the PDQ-39 reflect poorer quality of life. These two surveys have previously been validated in PD [18] and elderly adults [19]. To quantify balance confidence, participants completed the Activities and Balance Confidence (ABC) scale, a 16-point questionnaire in which participants are asked about their balance confidence during certain activities [20]; high scores indicate greater balance confidence. Finally, participants answered several questionnaires regarding their attitudes and beliefs about exercise, including confidence about ability to maintain an exercise program (CONF), exercise control beliefs (BEL) [21], and the self-efficacy for exercise scale (EFFIC) [22]. High scores are associated with greater confidence about exercise programs (CONF), more negative beliefs about exercise (BEL), and positive exercise self-efficacy (EFFIC). 

Outcome variables, which included SELF, FAST, and DIFF (i.e., FAST-SELF), were calculated based upon timed 10-meter walks on a straight path. For each walk, participants were given a 2-meter initiation and termination phase for a total walking distance of 14 meters; walking speed was measured for the 10-meter distance between the initiation and termination phases. For SELF, participants were instructed to walk at a comfortable pace after “Ready” and “Go” cues. For FAST, participants were told to walk as quickly and safely as possible after “Ready” and “Go” cues. Participants always started with SELF and performed one trial at each speed. Gait speeds were normalized for subject height [23]. We performed analyses on both normalized and raw gait speeds and were met with similar results. As such, normalized gait speeds were used for all subsequent analyses.

### 2.3. Regression Model Selection

To determine the significant predictors of SELF, FAST, and DIFF gait speeds, we used block-entry linear regression. Potential variables for inclusion in the regression were first determined using bivariate analyses. A variable was removed from consideration in the regression analysis if its Pearson correlation coefficient with any of SELF, FAST, or DIFF gait speed was less than 0.25. This cut-off criterion ensured that each variable would explain a minimum of 6% of the variability in gait speed, not accounting for collinearity. After filtering out unrelated variables, we ensured that all measures were not different between men and women. For variables in which there were differences between men and women (DIFF, see Results), a separate gender-stratified regression model was developed. All predictor variables were rescreened for this model using a bivariate coefficient cutoff of 0.35 (to account for reduction in the sample size). 

After filtering variables with low correlation to gait speeds, we constructed the models using a hybrid approach. *A priori* predictors age, and MDS-UPDRS were selected first based on previous reports describing the relationships between gait performance, age and disease severity [10, 24, 25]. The entire MDS-UPDRS score (sum of all four subscales) was used because it includes motor and nonmotor aspects of the disease as well as activities of daily living and motor complications. Our hypothesis-driven predictors included ABC, GDS, PDQ, and three exercise-attitude scales (BEL, CONF, and EFFIC). 

Table 1 defines the blocks and included variables for each regression model predicting SELF, FAST, and DIFF. We grouped predictors into the following blocks: (1) age, (2) disease severity (MDS-UPDRS); (3) balance confidence (ABC); (4) quality of life/mood (PDQ-39 and GDS); (5) attitudes about exercise (CONF, BEL, and EFFIC). The blocks for the stratified analysis of DIFF were as follows: (1) disease severity (MDS-UPDRS); (2) balance confidence (ABC), (3) quality of life (GDS and PDQ), and (4) attitudes about exercise (BEL). Age, CONF, and EFFIC were not included in the gender-stratified DIFF model because they did not meet the inclusion criterion (*r* > 0.35) for both men and women. We evaluated each model based on the adjusted *R* ^2^ change and the change in overall model *F* statistic after the addition of each block and by the statistically significant predictors in the final model. 

### 2.4. ROC Analyses

To determine if gait speed could discriminate among fallers in our sample, we generated receiver operator characteristic (ROC) curves and calculated area under the curve (AUC), cut-off scores, sensitivity, specificity, and likelihood ratios (see [26]) for SELF, FAST, and DIFF speeds, and also for DIFF separately in men and women. Cut-off scores were calculated by:

(1) $$\begin{array}{c} \frac{\ln\left(p/\left(1-p\right)\right)-a}{B}*h, \end{array}$$

where *p* is the probability of falling associated with the maximum sensitivity and specificity; *a* is the intercept coefficient from the logistic regression of normalized gait speed to predict falling; *B* is coefficient of regression; and *h* is the mean height (m). Fall data were taken from a questionnaire inquiring how often the participant had fallen in the past 6 months. A fall was described as an unexpected event in which any part of the body contacted the ground [27, 28]. Fallers were defined as those who fell two or more times over the past 6 months as assessed via self-report. As such, in our study, fallers were categorized as recurrent fallers. This criterion ensured that we could distinguish actual fallers who have significant gait and balance impairment from those who fell randomly [29]. 

All statistical analyses were performed using SPSS version 20 (IBM, Chicago, IL, USA). Differences in variables between genders were determined using independent sample *t*-tests or Mann-Whitney *U* tests for categorical or nonnormally distributed data. The level of significance was set at *α* = 0.05 unless otherwise noted. 

## 3. Results

Participant demographics and experimental variables are shown in Table 2 for men, women, and the total sample. The average SELF gait speed was 1.10 m/s, while the average fast-as-possible gait speed was 1.53 m/s. DIFF was significantly greater in men compared to women (0.51 ± 0.28 m/s versus 0.32 ± 0.18 m/s; independent sample *t*-test; *P* < 0.001) after controlling for height differences. All other measures were not different between men and women.

Table 3 summarizes the regression model predicting SELF, FAST, and DIFF gait speeds in the total sample. The model was able to predict 51.9% and 54.1% of the variation in SELF and FAST, respectively, while the same model only accounted for 20.2% of the variability in DIFF. Blocks 1–3 (age, disease severity, and balance confidence) were significant contributors to the model predicting SELF and DIFF, while all except block 5 (attitudes about exercise) made significant contributions in explaining FAST. Despite the overall model potency, only age and ABC were significant predictors of SELF and FAST after accounting for all other variables. There were no significant regressors in the final model of DIFF. 

After noting gender differences in DIFF, we created a separate model to describe DIFF in men and in women.  Table 4 shows the model results for DIFF in men and women using four blocks. Overall, the model accounted for 20.7% of the variability in men's DIFF walking speed. Block 1 (disease severity) was a significant contributor while block 3 (mood/quality of life) was marginally significant (*P* = 0.066). For women, only block 1 (disease severity) was a significant block (*P* = 0.012). Overall, 15.8% of the variability in DIFF walking speed was explained by the model for females, which was not significantly different (*P* = 0.084) from the null model, that is, a model without any predictor variables. Moreover, the addition of block 3 (mood/quality of life) reduced the potency of the model, as shown in the relatively large and negative change in *R* ^2^. 

**Figure 1:**
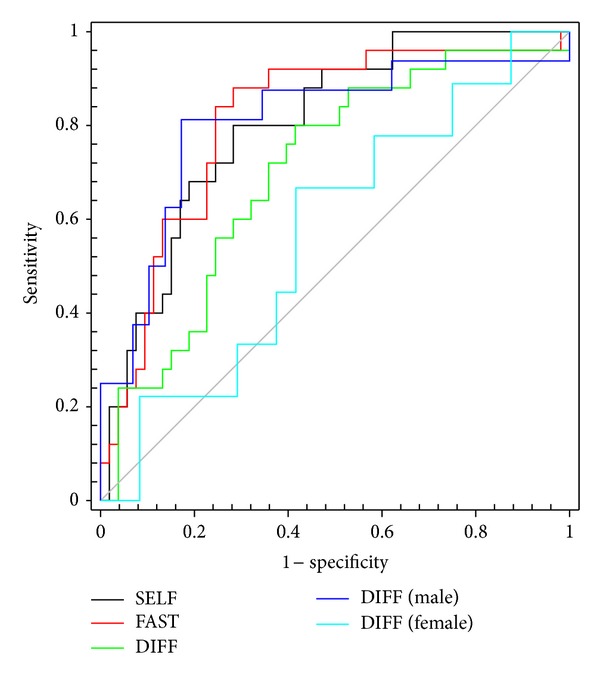
ROC curves for SELF (black), FAST (red), and DIFF (green) predicting fallers in the total sample. DIFF was also used to predict fallers, respectively, in men (blue) and women (cyan). SELF and FAST were strong predictors of fallers in the pooled sample. DIFF was a strong predictor of fallers in men but not women (see Table 5 for AUC, sensitivity, and specificity values).

To determine the value of gait speed alone to discriminate among fallers and nonfallers, we generated ROC curves (Figure 1) for SELF, FAST, and DIFF in the total sample.  Table 5 shows the AUC, cut-off score, sensitivity, specificity, and likelihood ratios associated with each test. SELF (*P* < 0.001), FAST (*P* < 0.001), and DIFF (*P* = 0.004) were all significantly associated with fallers in the total sample, according to the AUC. Due to the aforementioned gender differences, we further investigated if DIFF was a better predictor of fallers in men or women (Figure 1). DIFF was a strong predictor of male fallers (AUC = 0.806, *P* = 0.001; sensitivity = 0.828; specificity = 0.813) but a relatively weaker predictor of female fallers (AUC = 0.569, *P* = 0.544; sensitivity = 0.667; specificity = 0.583).

## 4. Discussion

In this work, we identified significant non-gait-related predictors of comfortable, fast-as-possible, and the difference between these walking speeds in individuals with PD. We showed that age, disease severity, and balance confidence were significantly related to all three gait speeds. Furthermore, we showed how gait speeds were significantly associated with a history of falls in the past 6 months. Gender-stratified analyses indicated that DIFF was well explained by disease severity and in part by mood/quality of life in men, but not in women. 

We determined that age, disease severity (i.e., MDS-UPDRS), and balance confidence (i.e., ABC) were important predictors of SELF, FAST, and DIFF gait speeds. When considering both genders together, our data expanded upon previous research [7, 8] identifying age and disease severity not only as predictors of SELF, but also as predictors of FAST and DIFF. In addition, although we had hypothesized that nonmotor factors (e.g., low balance confidence) would influence DIFF, our data suggested that balance confidence is significantly related to overall gait performance in people with PD. This finding was in agreement with other studies examining the relationship between ABC and gait [30, 31] but was not surprising, given that several items on the ABC pertain to everyday gait activities such as walking around the house. 

Our model was able to explain about 2.5 times more variability in SELF and FAST compared to DIFF, indicating that DIFF may not be as clinically useful compared to SELF and FAST when assessing people with PD. Blocks 3–5 (ABC, mood/quality of life, attitudes about exercise) only contributed an additional 0.075 to the *R* ^2^ value in the DIFF model after controlling for age and disease severity, compared to 0.15 and 0.178 *R* ^2^ increases for the SELF and FAST models, respectively. It is possible that DIFF was actually measuring disease severity, meaning that lower DIFF represented a physical inability to increase speed, rather than a lack of internal drive. However, DIFF was weakly correlated with UPDRS scores (*r* = −0.365) compared to SELF (*r* = −0.575) or FAST (*r* = −0.574). Alternatively, some of the variance in DIFF could have resulted from an unidentified interaction of two or more variables, which if it occurred, would confound the relationship between nonmotor behavior and change in gait speed. Interestingly, the block representing mood/quality of life significantly contributed to the variation in FAST but not SELF gait speed (Block 4, Table 3); that is, those who have a greater maximum gait speed may have better mood and quality of life. We were limited in our ability to interpret this finding because we used the total PDQ-39 summary score and did not analyze each subsection (emotion, cognition, stigma, etc.). The scores of a certain sub-section may in fact have driven the differences, but subsection analysis was beyond the scope of this paper. Overall, the regression models did not reveal a major distinction in the characteristics of an individual based on his/her SELF and FAST walking speed. Future work should consider longitudinal analyses of gait speed given that gait speed predicts declines in attention and psychomotor abilities over time in older adults [32].

Research focusing on identifying fallers and predicting falls is important in order to reduce injury and prevent future falls in PD. Many groups have used a variety of gait and balance characteristics to predict fallers with much success [33–36]. Here we showed that, by themselves, SELF, FAST, and DIFF (in men only) were strongly associated with falling in people with PD. This finding is in contrast to Duncan and Earhart [37], who determined that SELF and FAST gait speeds were poor predictors of falling in a PD cohort compared to balance assessments. One major distinction between that work and the present study is that Duncan and Earhart measured participants off medication. We assessed participants on medication, which has been shown to improve self-selected gait speeds [38, 39]. Given that people are normally medicated during daily activities, assessments conducted on medication may be better at predicting fallers compared to off-medication assessments. In any case, our AUC values from SELF and FAST speeds were comparable to other balance and gait measures for fall prediction [34, 35]. 

Our ROC results were in line with Paul et al., who determined a self-selected gait speed cutoff of 1.1 m/s to help predict future fall risk [14]. Our cut-off value (0.98 m/s) was slightly more stringent. Despite this minor difference, together these findings supported the use of gait speed for screenings to predict future fallers. These screenings would complement existing evaluation tools such as multi-item balance assessments and 3-dimensional gait kinematics. Balance assessments require trained raters and longer evaluation times, while kinematic measurements need sophisticated equipment and analysis techniques [4, 5]. In contrast, gait speed can be quickly and accurately measured in any setting with only a stop-watch and measuring tape and a minimally trained rater.

Our data indicated that men increased their gait speed from SELF to FAST more than women, even when corrected for height. Consequently, we evaluated DIFF in men and women using a second hierarchal regression model and also determined if this speed differentially predicted falls in men and women. Our model explained only 5% more variability in DIFF in men compared to women. This marginal distinction between genders was primarily due to a 7% change from block 2 to 3, showing a contribution from the block representing mood/quality of life in men (Table 4). However, DIFF accurately identified male but not female fallers. This result lends some support for the value of DIFF when assessing male PD patients. However, DIFF does not provide additional information over SELF or FAST to predict male fallers, and thus its value in screening is not clear. While some work has investigated gender differences in PD disease severity and motor symptoms [40, 41], to date no one has shown any gait-related gender differences. Additional studies examining gender differences in gait characteristics may help define the relationship between gait speed and falling. 

There are several limitations of our work that should be addressed. First, we examined this sample at only one time point, which may limit the relationships between gait speed, disease severity, and nonmotor behavior. A longitudinal analysis would allow us to detect individual changes in these variables over time and may help reduce intrasubject variability. Furthermore, we did not randomize the order of SELF and FAST which may have induced a priming effect on FAST speeds. Secondly, in our model selection, we tried to choose both statistically relevant and sensible predictors of gait speed. In doing so, we did not include terms that, while not significantly correlated with gait speed, may have added to our overall model potency. To add, many predictor variables were highly intercorrelated, and while our collinearity diagnostics for either model returned no major concerns, this may have influenced individual predictor strength and final model interpretation. Finally, we collected fall data via subject recall. Hannan et al. showed that older individuals may underestimate how many times they fell in the last three months when measured with self-report compared to tracking falls with a calendar [42]. As such, our study may have lacked sufficient power to discriminate fallers from nonfallers.

## 5. Conclusion

Using a blockwise linear regression model, we identified age, disease severity, and balance confidence to be strong predictors of comfortable and fast-as-possible gait speeds in a cohort of people with PD. SELF and FAST were also significantly related to fallers in our total sample, illustrating the potential utility of including gait speed as a screening measure for individuals with fall risk. The difference between SELF and FAST gait speeds was not well described by nonmotor behavior in the total sample. However, mean DIFF speed was larger and more associated with fallers in men than in women, suggesting that it may be a more informative measure in men. 

## Figures and Tables

**Table 1:** Regression model specification.

Block	Predictor variable(s)	Outcome variable(s)
(1) Age	Age	SELF, FAST, DIFF
(2) Disease severity	MDS-UPDRS	SELF, FAST, DIFF
(3) Balance confidence	ABC	SELF, FAST, DIFF
(4) Mood/quality of life	GDS PDQ	SELF, FAST, DIFF
(5) Attitudes about exercise	BEL CONF EFFIC	SELF, FAST, DIFF

(1) Disease severity	MDS-UPDRS	DIFF M/F
(2) Balance confidence	ABC	DIFF M/F
(3) Mood/quality of life	GDS PDQ	DIFF M/F
(4) Attitudes about exercise	BEL	DIFF M/F

**Table 2:** Participant demographics and experimental variables.

Characteristic (scale)	Total	Males	Females	*P* (M versus F)
Sex, *N*	78	45	33	—
Age, yr	68.18 ± 9.35 (45, 88)	67.84 ± 8.84 (48, 88)	68.63 ± 10.12 (45, 85)	0.714
Disease duration, yr	8.50 ± 4.88 (0, 25)	8.82 ± 5.33 (0, 25)	8.06 ± 4.22 (1, 20)	0.499
Fallers, *N* (% total)	25 (32%)	16 (36%)	9 (27%)	0.439^d^
MDS-UPDRS (0–260)	72.96 ± 24.99 (25, 135)	74.78 ± 26.71 (26, 131)	70.48 ± 22.60 (25, 135)	0.457
MDS-UPDRS-III (0–132)	41.52 ± 14.77 (9, 83)	43.02 ± 14.30 (17, 70)	39.48 ± 15.37 (9, 83)	0.299
H&Y^a^ (0–5)	2.50 (0.63)	2.50 (0.5)	2.50 (1.0)	0.473^c^
FOGQ (0–24)	6.97 ± 5.85 (0, 20)	6.78 ± 5.82 (0, 20)	7.24 ± 5.97 (0, 19)	0.731
GDS (0–30)	8.47 ± 6.28 (0, 24)	8.87 ± 6.42 (0, 24)	7.93 ± 6.15 (0, 24)	0.523
PDQ-39 (0–100)	22.83 ± 13.72 (0.52, 63.13)	22.05 ± 15.00 (0.52, 66.13)	23.91 ± 11.90 (2.86, 51.77)	0.558
FGA (0–30)	18.41 ± 7.10 (0, 29)	19.29 ± 6.99 (4, 29)	17.21 ± 7.20 (0, 29)	0.204
ABC (0–100)	68.27 ± 25.01 (16, 100)	72.51 ± 23.87 (17, 100)	62.48 ± 25.73 (16, 100)	0.08
BEL (0–25)	8.19 ± 2.96 (5, 16)	8.22 ± 3.14 (5, 16)	8.15 ± 2.74 (5, 15)	0.838^c^
CONF (0–40)	28.21 ± 8.66 (8, 40)	29.71 ± 7.90 (8, 40)	26.18 ± 9.35 (9, 40)	0.08^c^
EFFIC (0–10)	5.48 ± 2.26 (0.33, 9.44)	5.62 ± 2.38 (0.78, 9.44)	5.30 ± 2.12 (0.33, 8.89)	0.536
SELF, m/s	1.10 ± 0.29 (0.37, 1.66)	1.15 ± 0.29 (0.49, 1.67)	1.04 ± 0.29 (0.37, 1.44)	0.822^b^
FAST, m/s	1.53 ± 0.47 (0.54, 2.80)	1.66 ± 0.49 (0.67, 2.80)	1.36 ± 0.40 (0.54, 2.18)	0.104^b^
DIFF, m/s	0.43 ± 0.26 (−0.05, 1.27)	0.51 ± 0.28 (0.04, 1.27)	0.32 ± 0.18 (−0.05, 0.74)	**0.008** ^ b^

^a^Data presented as median (IQR).

^b^ *P* values represent differences in normalized gait speed.

^c^Mann-Whitney *U* test; ^d^Chi-square test.

All other data presented as mean ± SD (Min, Max).

Gender differences were determined by independent samples *t*-test unless otherwise noted.

MDS-UPDRS: Movement Disorder Society Unified Parkinson Disease Rating Scale; H&Y: Hoehn and Yahr stage; FOGQ: Freezing of Gait Questionnaire; GDS: Geriatric Depression Scale; PDQ-30: Parkinson Disease Questionnaire-39; FGA: Functional Gait Assessment; ABC: Activities and Balance Confidence Scale; BEL: Beliefs about control over one's exercise behavior; CONF: confidence about maintaining an exercise program; EFFIC: self-efficacy exercise scale; SELF: self-selected gait speed; FAST: fast-as-possible gait speed; DIFF: difference between FAST and SELF.

**Table 3:** Regression model results for SELF, FAST, and DIFF walking speeds.

Block	Variable	*B*_unstd_	*B*_std_	*P*	*R* ^2^ change	*P* (block)
SELF						

1	Age	−0.005	−0.277	**0.004**	0.189	<**0.001**
2	UPDRS	0.001	0.016	0.906	0.18	<**0.001**
3	ABC	0.003	0.448	**0.001**	0.155	<**0.001**
4	GDS	−0.002	−0.092	0.411	0.002	0.328
	PDQ-39	−0.002	−0.146	0.349		
5	CONF	0.003	0.144	0.228	−0.007	0.565
	BEL	0.001	0.021	0.851		
	EFFIC	−0.009	−0.117	0.258		

					Total *R* ^2^ = 0.519**	

FAST						

1	Age	−0.008	−0.288	**0.002**	0.179	<**0.001**
2	UPDRS	0.001	0.083	0.529	0.184	<**0.001**
3	ABC	0.004	0.342	**0.009**	0.146	<**0.001**
4	GDS	−0.004	−0.085	0.438	0.035	**0.026**
	PDQ-39	−0.005	−0.283	0.065		
5	CONF	0.004	0.124	0.286	−0.003	0.478
	BEL	−0.006	−0.063	0.554		
	EFFIC	−0.009	−0.079	0.430		

					Total *R* ^2^ = 0.541**	

DIFF						

1	Age	−0.003	−0.196	0.109	0.057	**0.02**
2	UPDRS	0.001	0.129	0.458	0.07	**0.01**
3	ABC	0.001	0.098	0.564	0.045	**0.028**
4	GDS	−0.001	−0.046	0.750	0.042	0.058
	PDQ-39	−0.004	−0.336	0.095		
5	CONF	0.001	0.057	0.710	−0.012	0.584
	BEL	−0.007	−0.136	0.337		
	EFFIC	−0.001	−0.008	0.951		

					Total *R* ^2^ = 0.202*	

**P < 0.001, *P < 0.05 overall model is significant.

*B*_unstd_: unstandardized coefficient; *B*_std_: standardized coefficient.

**Table 4:** Regression model results for DIFF in men and women.

Block	Variable	*B*_unstd_	*B*_std_	*P*	*R* ^2^ change	*P* (block)
Men						

1	UPDRS	−0.001	−0.086	0.722	0.143	**0.006**
2	ABC	−0.001	−0.147	0.557	−0.016	0.635
3	GDS	−0.005	−0.184	0.411	0.073	0.066
	PDQ	−0.003	−0.296	0.265		
4	BEL	−0.010	−0.196	0.253	0.007	0.253

					Total *R* ^2^ = 0.207*	

Women						

1	UPDRS	−0.001	−0.254	0.366	0.162	**0.012**
2	ABC	0.001	0.279	0.271	0.045	0.107
3	GDS	0.001	0.006	0.974	−0.055	0.978
	PDQ	0.001	0.068	0.832		
4	BEL	−0.008	−0.197	0.285	0.006	0.285

					Total *R* ^2^ = 0.158	

*P < 0.05 overall model is significant.

*B*_unstd_: unstandardized coefficient; *B*_std_: standardized coefficient.

**Table 5:** AUC, specificity, sensitivity, and LR for ROC curve analyses of gait speeds.

Measure	AUC (95% CI)	Cut-off score (m/s)	Sensitivity	Specificity	LR+ (95% CI)	LR− (95% CI)
SELF	0.803 (0.704, 0.902)	0.980	0.800	0.717	2.827 (1.765, 4.528)	0.279 (0.125, 0.622)
FAST	0.811 (0.707, 0.916)	1.326	0.840	0.755	3.429 (2.072, 5.659)	0.212 (0.085, 0.527)
DIFF	0.703 (0.581, 0.826)	0.226	0.720	0.642	2.011 (1.300, 3.104)	0.436 (0.226, 0.845)
DIFF (male)	0.806 (0.659, 0.953)	0.356	0.813	0.828	4.727 (2.052, 10.822)	0.226 (0.806, 0.637)
DIFF (female)	0.569 (0.356, 0.783)	−0.067	0.667	0.583	1.600 (0.826, 3.100)	0.571 (0.214, 1.529)
